# Four Botanical Extracts are Toxic to the Hispine Beetle, *Brontispa longissima,* in Laboratory and Semi—field Trials

**DOI:** 10.1673/031.012.5801

**Published:** 2012-05-02

**Authors:** Lv Chaojun, Zhong Baozhu, Zhong Guohua, Weng Qunfang, Chen Shaohua, Hu Meiying, Sun Xiaodong, Qin Weiquan

**Affiliations:** ^1^ Coconut Research Institute, Chinese Academy of Tropical Agricultural Sciences, Wenchang, Hainan 571 339, China; ^2^ Key Laboratory of Natural Pesticide and Chemical Biology (Ministry of Education), South China Agricultural University, Guangzhou 510642, China

**Keywords:** antifeedant activity, oral toxicity, ovicidal activity

## Abstract

The potential of botanical extracts such as *Celosia argenea* L. (Caryophyllales: Amaranthaceae), *Ricinus communis* L. (Malpighiales: Euphorbiaceae), *Mikania micrantha* Humboldt, Bonpland & Kunth (Astrales: Asteraceae), and *Catharanthus roseus* (L.) G. Don (Gentianales: Apocynaceae) for the control of *Brontispa longissima* Gestro was evaluated in a bioassay and semi—field trial. Dose—response bioassay showed no significant difference in oral—toxicity among the extracts of *C. argenea, M. micrantha,* and *C. roseus* to larvae and adult of *B. longissima.* All extracts tested decreased the hatchability of *B. longissima* eggs. In particular, the extract of *M. micrantha* showed higher activity than others at the concentration of 5 mg/mL. In an antifeedant bioassay, the extract of *C. argenea* showed higher activity against the 1^st^ larvae than that of other extracts (AF50 0.03 mg/mL), and *C. roseus* showed higher antifeedant activity to the 2^nd^ to 5^th^ larvae and adult of *B. longissima* (AF50 0.34, 0.33, 0.11, 0.43, and 0.20 mg/mL, respectively). The semi—field trial indicated that all extracts used in this study might reduce the pest population. Extracts of *C. argenea* and *M. micrantha* showed higher activities than that of *C. roseus* and *R communis,* and the decrease in population was 75.56% and 80.00% (without Abbott's correction) after seven days of treatment, respectively, at a concentration of 20 mg/mL. Therefore, these active botanical extracts may possess potential for use in control of *B. longissima.*

## Introduction

The coconut hispine beetle, *Brontispa longissima* Gestro (Coleoptera: Chrysomelidae), is a major pest of coconut palm. This pest is native to Indonesia and Papua New Guinea, and widespread in Southeast Asia, Australia, Solomon Islands, and Pacific regions (Hassan 1972; Stapley 1973, 1980; Jones 1986). *B. longissima* is also a quarantined insect in China (Lu 2004), where it was introduced in 1975 (Cheng 1991; Lau 1991). In 2002, *B. longissima* was first discovered in Hainan province of China and caused considerable damage to palm plants, especially the coconut. The palms in Guangdong, Guangxi, Yunnan, and Fujian provinces of China were also threatened (Lu 2004). Chemical pesticides are presently the most widely used control method for *B.* longissima, causing widespread damage to the environment and natural enemies. Moreover, insecticide resistance has developed in *B. longissima* (Miao 2005), therefore, new methods should be investigated. Plants may serve as this critical resource. In addition to their production of defensive substances with co—evolutionary origins (Kessler 2002; Nina 2003; Consuelo 2004), their botanical extracts are generally considered to be safe for human and animals, and have a variety of control mechanisms (Sivagnaname 2004). Therefore, effects of four botanical extracts on *B. longissima* were studied in order to evaluate their control potential and provide a new approach of Integrated Pest Management (IPM) of *B. longissima.*

## Materials and Methods

### Insects

Specimens of *B. longissima* were collected from the field in Wenchang and Haikou municipalities of Hainan province, China, and reared for at least five generations on core coconut leaves. The insects were maintained in environmental conditions of 25 ± 1 ^°^C and 75–80% relative humidity, with 14:10 L:D photoperiod.

### Plant materials

*Celosia argenea* L. (Caryophyllales: Amaranthaceae), *Ricinus communis* L. (Malpighiales: Euphorbiaceae), *Mikania micrantha* Humboldt, Bonpland & Kunth (Astrales: Asteraceae), and *Catharanthus roseus* (L.) G. Don (Gentianales: Apocynaceae) were obtained from Wenchang municipalities of Hainan province, China. Leaves were collected and dried at 60 ^°^C in an incubator for two days and then ground into a powder. Three grams of ground powders were placed in containers with 100 mL methanol. In order to prevent solvent evaporation, the containers were covered with parafilm and foil and held in a refrigerator for 24 hours. After being filtered through a 0.22µm Millipore filter (Millipore, www.millipore.com), the extracts were dried to powder using a rotary evaporator under reduced pressure at 45 ^°^C to remove the solvent (Fliniaux 2004). The extracted powders were stored in a refrigerator. Extract powders were diluted with acetone for laboratory bioassays or in distilled water for semi—field trials.

### Oral toxicity bioassay

The oral toxicity of botanical extracts on larvae or adults of *B. longissima* were investigated using a leaf dipping method (Shukla 2010). The developing core leaves of coconut were cut (10 cm length) and immersed into 10 mL of extract solutions using acetone as the solvent (5–50 mg/mL) for 10 minutes, and then removed and dried for two hours under air flow in a chemical hood. After drying, sets of 30 different instar larvae or adults of *B. longissima* were placed onto each treated coconut leaf, and then placed in a glass tube (150 mm length, 15 mm diameter). Leaves dipped in 10 mL acetone were used as control. For each treatment, three replicates were conducted. Percentage mortality was recorded after 72 hours; insects that showed no response with head movements or exhibited peristaltic contraction when touched with a fine brush were scored as dead. The larvae that had molted into pupa were scored as alive.

### Ovicidal bioassay

To determine the toxicity of different extracts on *B. longissima* eggs, males and females emerging from the laboratory colony were maintained for 10 days in a plastic mating cage (10 × 8 cm) with developing leaves of coconut as food. One female and two males were then removed from the mating cage and transferred to an ovipositor chamber consisting of a cylinder (15 cm length, 2 cm diameter) covered with a # 200 nylon screen. After eggs were deposited, coconut leaves with 30 eggs were dipped into extract solutions using acetone as the solvent (5 mg/mL) for five sec and then removed and placed under a chemical hood to dry for two hours. Coconut leaves with 30 eggs dipped into acetone were used as control. Three replicates of each treatment were conducted, and all replications were performed at the same time. The mortality ratio was calculated until no more eggs hatched.

### Antifeedant bioassay

The bioassay was performed according to the method developed by Omar et al (2007). Extract was diluted by acetone and concentration ranged from 0.5 to 5 mg/mL, with acetone acting as the control. Tender coconut leaves (10 cm length) were immersed into 10 mL of extract solution for 10 minutes, then removed and dried for two hours under air flow in a chemical hood. Leaves immersed with acetone were used as controls. After drying, 10 larvae or 10 adults of *B. longissima* were placed onto each treated coconut leaf, and then placed in a glass tube (150 mm length, 15 mm diameter). The remaining leaves were removed after 24 hours to calculate the insect's food consumption on a dry weight basis. Initial dry weight of leaves was estimated from the regression of fresh weight to dry weight of additional leaf disks. For each treatment, three replicates were performed.

### Semi—field trial

The semi—field trial was conducted at the Coconut Research Institute, Chinese Academy of Tropical Agricultural Sciences, China. The experimental designs were randomized complete blocks. All the plants were grown using the same cultural practices, including drip irrigation and fertilization as needed. The experiment started in early July 2010. There were five treatments (with one control) with six replications per treatment and six plants per replication. Coconut palms 2–3 meters high were chosen for use.

Botanical extracts at a dose of 20 mg/mL diluted with distilled water were used in the semi—field trial, whereas distilled water was used as the control. The treatments were sprayed by a manual sprayer (250 mL per tree). Once the applied extract solutions were dried, the core tender leaves were infested with 30 third instar larvae of *B. longissima.* In order to prevent the interference of escape and bodily injuries caused by experimental manipulations, the pest number was kept at 30 during the first six hours, as pre—test trials indicated that six hours was insufficient for extracts to act. Finally, after 1, 3, 5, and 7 days, the treatment effects were assessed by counting the number of surviving specimens.

### Statistical analysis

Egg, larvae, and adult percentage mortality were analyzed, and Abbott's formula (Abbott 1925) was applied to correct percentage mortality if control mortality was between 5% and 20%.


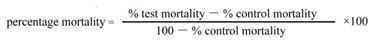


The Probit Regression program in Statistical Package for Social Science (SPSS) version 12 was used to determine LC-P line, lethal concentration values, and corresponding 95% fiducial limits of the upper confidence limit and the lower confidence limit. Significant difference was determined by non— overlapping of the 95% confidence limit.

In the semi—field trial, the percentage mortality was corrected through Abbott's formula, and the results were analyzed with Duncan's new multiple range test using Statistical Analysis System (SAS) version 8.1. *B. longissima* that escaped were counted as alive.

## Results

### Larvicidal and adulticidal bioassays

The response of *B. longissima* larvae and adults exposed to different botanical extracts is shown in Table 1. The results indicated that extracts of *C. argenea, M. micrantha, C. roseus,* were toxic to *B. longissima* except for the *R communis* extract, which showed significantly lower toxicity.

No significant difference was shown in toxicities of *C. roseus* and *R. commnuis* extract to different instar larvae and adults of *B. longissima,* based on 95% confidence limits of LC_50_.

### Ovicidal bioassay

All botanical extracts used displayed bio—activity toward *B. longissima* eggs by decreasing hatchability (Table 2). The hatching rate of normal treatment (control) was 93.33% in six days. After treated with the extracts of *C. argenea, M. micrantha, C. roseus,* and *R. communis,* the hatchability decreased. Among the extracts used, *M. micrantha* showed the highest bio—activity.

### Antifeedant bioassay

The antifeedant effects of botanical extracts on larvae and adults of *B. longissima* are shown in Table 3. The results clearly indicated that the extract of *C. argenea* showed higher antifeedant effect on 1^st^ larvae with AF50 0.03 mg/mL, followed by *M. micrantha* and *C. roseus.* However, the extract of *C. roseus* showed higher antifeedant activity to 3^rd^ instars and adults of *B. longissima,* and similar antifeedant effects occurred in 4^th^ and 5^th^ instars between extracts of *C. roseus* and *C. argenea,* as well as in 2^nd^ instars of *B. longissima.* There was a significant difference between the antifeedant effects of *C. roseus* and *M. micrantha* to different stages of *B. longissim,* except among 2^nd^ instars. Extract of *R. communis* showed the lowest antifeedant effects in instars of *B. longissima* compared with others.

**Table 1.**
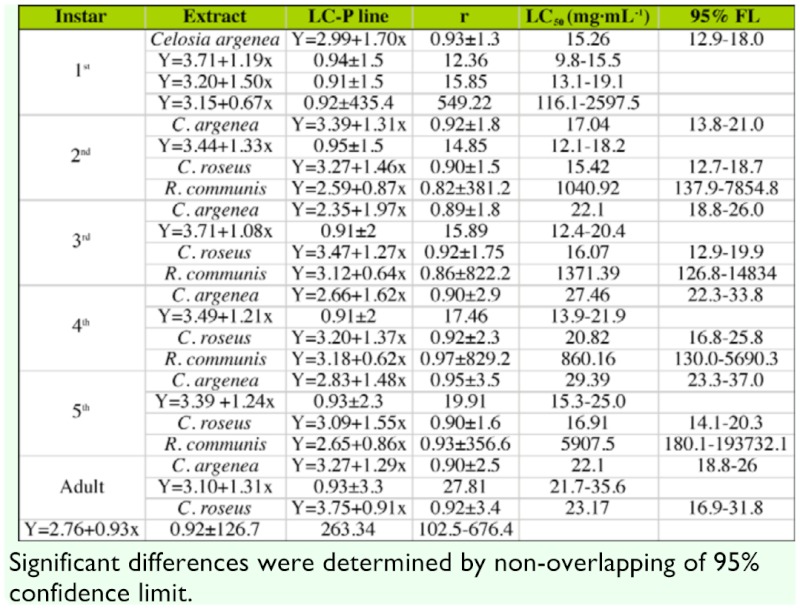
Lethal concentration values (LC_50_, mg·mL-^1^) for oral toxicity of different extracts to larvae and adults of *Brontispa longissima,* assessed in laboratory with dipping test.

**Table 2.**
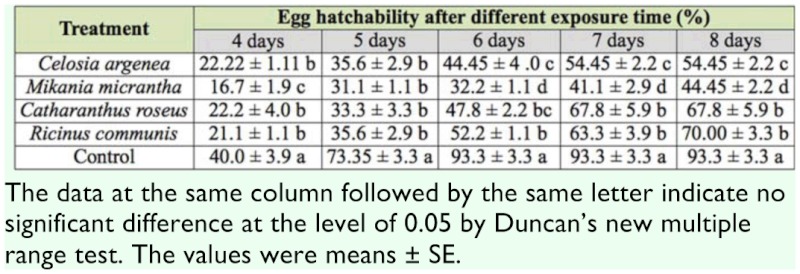
Effect of different extracts to egg hatchability of *Brontispa longissima,* assessed in laboratory with a dipping test.

**Table 3.**
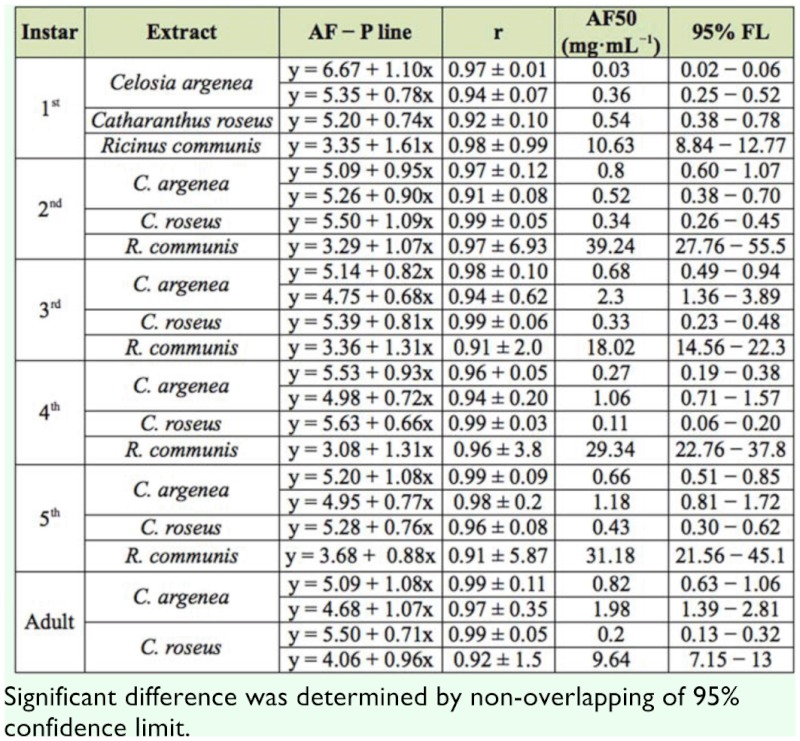
Dose response for antifeedant effect of different extracts to *B*. *longissima,* assessed in laboratory with dipping test.

### Semi—field trial

The total pest population decreased after treatment with the botanical extracts at the concentration of 20 mg/mL (Table 4). Extracts of *C. argenea* and *M. micrantha* showed higher control efficiency than that of *C. roseus* and *R communis.*

## Discussion

An acceptable insecticide effective kills target organisms, but results in low mortality of non—target organisms. Phytochemicals may serve as suitable alternatives to synthetic insecticides in future because they are relatively safe, inexpensive, and readily available throughout the world. Qin et al. (2010) documented that alcohol extracts of *Piper sarmentosum* and *Artocarpus heterophllus* could slow down the development of *B. longissima;* the generation cycle of *B. longissima* was found to be 73.58 days when treated with 0.01 g/mL of *P. sarmentosum* extract, which was delayed compared with the control (43.34 days). Additionally, the fecundity of *B. longissima* declined in the same study. The essential oil from *P. sarmentosum* had strong antifeedant and oral toxicity on *B. longissima,* and our results in the test indicated that all four botanical extracts showed oral toxicity to *B. longissima* from 1^st^ instar to adult, especially extracts of *C. argenea, M. micrantha,* and *C. roseus.* All botanical extracts used may decrease hatchability; some may even reduce egg hatchability by half, notably the *M.* *micrantha* extract. This could prove extraordinarily effective in the control of *B. longissima* by reducing the pest population before they start to cause any damage. Furthermore, all extracts used showed antifeedant effects, which indicated the various uses of botanical extract in *B. longissima* control. These results confirmed the conclusions reported by Pang (2000) that plant secondary substances might have oral toxicity and antifeedant activity properties, and act to slow down development in pests. Though the required effective quantity of botanical extract is greater than that of synthetic insecticides, the preparation of suitable formulations using additives might enhance their efficiency. To our knowledge, these extracts have not been tested against other insects, and it is possible that the effects shown on *B. longissima* may extend to other insects. However, the full potential of the four extracts in reducing damage on the plant and controlling the pest remains unknown.

**Table 4.**
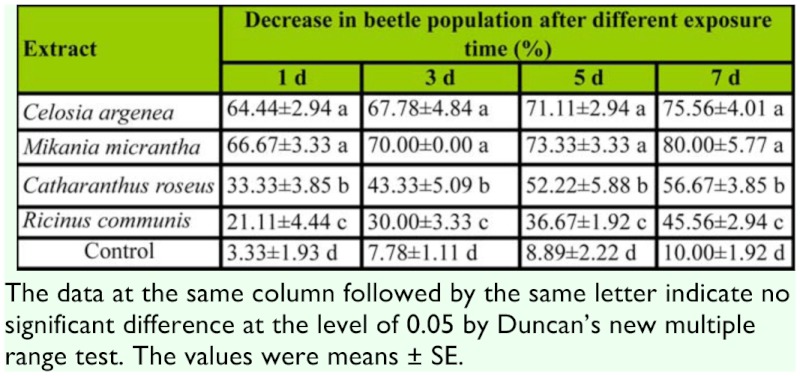
Effect of different extracts on *B*. *longissima* larvae in the semi-field trial.

It is also worth noting that botanical extracts of *C. argenea, M. micrantha,* and *C. roseus* showed higher oral toxicity than that of *R. communis,* and extracts of *C. roseus* showed better antifeedant activity than others, and higher effects in hatchability inhibition appeared in *C. argenea* and *M. micrantha* extracts. Semi—field trial results also indicated that extracts of *C. argenea* and *M. micrantha* showed higher activity in decreasing the *B. longissima* population. All of these differences were perhaps caused by the various secondary substances in different botanical extracts.

Because of the many negative effects of using chemical insecticides in the control of *B. longissima* and the shifting trend towards to integrated control, our results emphasize the importance of exploring the effectiveness of using botanically—derived materials as supplements to insecticides in *B. longissima* control. Field evaluations and research into their mode of action and effects on non—target organisms are currently underway. Our study only represents a preliminary screening of target plants, and detailed bioassay—guided phytochemical studies are needed to identify active constituents. The relative environmental safety of identified constituents needs to be studied, since the biological activity of these plant extracts might be due to various extract components including phenolics, terpenoids, and alkaloids, which are present in plants (Park 2000). These compounds may jointly or independently contribute to produce bio—activity against *B. longissima.* Further fractionation and compound isolation, particularly of the minor components of botanical extracts, will hopefully reveal a potent phytochemical or synergistic mixture that will be comparable to or even better than synthetic insecticides. In addition, only methanol was used as a solvent for extracting potential bioactive constituents, indicating that only the effects of relatively polar constituents were compared. The effects of *B. longissima* constituents extracted by other solvents needs to be studied.
